# Long-term immunity against yellow fever in children vaccinated during infancy: a longitudinal cohort study

**DOI:** 10.1016/S1473-3099(19)30323-8

**Published:** 2019-12

**Authors:** Cristina Domingo, Juliane Fraissinet, Patrick O Ansah, Corey Kelly, Niranjan Bhat, Samba O Sow, José E Mejía

**Affiliations:** a Robert Koch Institute, Highly Pathogenic Viruses (ZBS 1), Centre for Biological Threats and Special Pathogens, WHO Collaborating Centre for Emerging Infections and Biological Threats, Berlin, Germany; b Navrongo Health Research Centre and Research Laboratory, Navrongo, Ghana; c PATH, Seattle, WA, USA; d National Institute of Research on Public Health, Bamako, Mali; e Centre de Physiopathologie Toulouse-Purpan (CNRS, INSERM, Université Paul Sabatier), Centre Hospitalier Universitaire Purpan, Toulouse, France

## Abstract

**Background:**

A single dose of vaccine against yellow fever is routinely administered to infants aged 9–12 months under the Expanded Programme on Immunization, but the long-term outcome of vaccination in this age group is unknown. We aimed to evaluate the long-term persistence of neutralising antibodies to yellow fever virus following routine vaccination in infancy.

**Methods:**

We did a longitudinal cohort study, using a microneutralisation assay to measure protective antibodies against yellow fever in Malian and Ghanaian children vaccinated around age 9 months and followed up for 4·5 years (Mali), or 2·3 and 6·0 years (Ghana). Healthy children with available day-0 sera, a complete follow-up history, and no record of yellow fever revaccination were included; children seropositive for yellow fever at baseline were excluded. We standardised antibody concentrations with reference to the yellow fever WHO International Standard.

**Findings:**

We included 587 Malian and 436 Ghanaian children vaccinated between June 5, 2009, and Dec 26, 2012. In the Malian group, 296 (50·4%, 95% CI 46·4–54·5) were seropositive (antibody concentration ≥0·5 IU/mL) 4·5 years after vaccination. Among the Ghanaian children, 121 (27·8%, 23·5–32·0) were seropositive after 2·3 years. These results show a large decrease from the proportions of seropositive infants 28 days after vaccination, 96·7% in Mali and 72·7% in Ghana, reported by a previous study of both study populations. The number of seropositive children increased to 188 (43·1%, 95% CI 38·5–47·8) in the Ghanaian group 6·0 years after vaccination, but this result might be confounded by unrecorded revaccination or natural infection with wild yellow fever virus during a 2011–12 outbreak in northern Ghana.

**Interpretation:**

Rapid waning of immunity during the early years after vaccination of 9-month-old infants argues for a revision of the single-dose recommendation for this target population in endemic countries. The short duration of immunity in many vaccinees suggests that booster vaccination is necessary to meet the 80% population immunity threshold for prevention of yellow fever outbreaks.

**Funding:**

Wellcome Trust.

## Introduction

Yellow fever is a persistent public health problem and a growing concern in 34 African countries and 13 countries in the Americas.^1^ The re-emergence of yellow fever has led to the largest outbreak in Africa of the past 20 years: the Angola outbreak of December, 2015, connected with the 2016 outbreak in the neighbouring Democratic Republic of the Congo. During the outbreak that started in Brazil in 2016, the virus spread into areas that were not previously considered at high risk, including the densely populated periphery of the large cities of São Paulo, Rio de Janeiro, and Salvador de Bahia.

The evolving epidemiology of the disease and the expansion of at-risk areas have been associated with prolonged periods of increased rainfall and temperatures and with environmental perturbations arising from human activity (eg, deforestation, population movements, and changes in land use).^2^ The resurgence of yellow fever, however, has been attributed largely to lapses of continuous vaccination coverage and the waning of population immunity in areas of yellow fever transmission, which makes outbreaks more frequent.^3^ It is estimated that 393·7–472·9 million people will require vaccination^4^ to achieve the population immunity recommended by WHO for countries at risk.^1^

The vaccines against yellow fever are safe and efficacious and consist of live attenuated virus that is usually administered by subcutaneous injection. WHO guidelines advocate a single dose of vaccine for life-long protective immunity against yellow fever.^5^ In endemic countries, the vaccine is routinely given to infants at 9–12 months of age as part of the Expanded Programme on Immunization. The merits of one-dose vaccination at such early ages have not yet been supported by evidence that shows vaccine-elicited immunity to yellow fever persisting for many years in the absence of booster doses. Several studies have shown a decrease in seropositivity and antibody titres in vaccines over time, with 71–82% of adults seropositive 10 years or more after vaccination.^6^ Studies on the loss of immunity in adults done in non-endemic settings, where a boost of vaccine-induced immunity by later natural infections is unlikely, have reported that up to 30%–40% of individuals serorevert 5–10 years after vaccination.^6^ Children, however, showed lower seroconversion rates and titres than healthy adults and might lose immunity faster.^7^

**Research in context**

**Evidence before this study**

WHO has recommended a single lifetime vaccination against yellow fever since 2013, following published evidence of long-lasting immunogenicity of the YF-17D vaccine. The requirement for a booster dose every 10 years was accordingly removed from the International Health Regulations in 2016. A shortcoming of this policy change is the scarcity of information on the effective duration of protective immunity elicited in vaccinated infants, even though this age group constitute the main vaccination target in yellow fever-endemic countries. We consulted reference documents prepared by WHO and by the Centers for Disease Control and Prevention on yellow fever vaccination and the primary sources on the duration of yellow fever vaccine immunity surveyed from four review articles from 2013–16, including one meta-analysis and one systematic review. We also searched PubMed on May 18, 2018, with the expressions “yellow fever immunity” and “children” or “infants”, “yellow fever immunity” and “persistence”, and “yellow fever vaccine”, and did a reverse search on the Web of Science database, for articles citing three highly relevant research reports. We did not apply any language or date restrictions. Although the yellow fever vaccine can elicit lifelong immunity, studies in healthy adults have observed a time-dependent decrease in the proportion of seropositive individuals to 71–82% 10 years or more after vaccination, down from the proportion of more than 90% usually detected during the first year. Studies in non-endemic settings, where interference from circulating flaviviruses can be discounted, have reported a 30–40% decrease in vaccine-induced immunity 5–10 years after yellow fever vaccination. Infants, and children in general, exhibit a higher vaccine failure and lower immune responses after vaccination than adults, but previous studies on the outcome of yellow fever vaccination have focused mainly on adults or older children. Studies covering vaccinated infants have been limited to determining seroconversion 1–3 months after vaccination.

**Added value of this study**

We show a large decline in humoral immunity to yellow fever after 2–6 years in each group of children relative to earlier observations 4 weeks after vaccination. Proportions of seropositive children approximately halved, leaving large proportions of the study populations with a negative serostatus. These results address an important knowledge gap and are informative on the evolution of conferred immunity, in two different African settings endemic for yellow fever.

**Implications of all the available evidence**

Our findings argue for the one-dose-for-life guidelines to be reconsidered for individuals who receive yellow fever vaccination as infants. The long-term decline of humoral immunity suggests that a single dose of the vaccine, administered at 9 months of age, might not achieve a population immunity protective against yellow fever epidemics.

Longitudinal studies of vaccine immunogenicity are essential to determine the risk of vaccination failure in individuals and to verify whether the single-dose policy guarantees community-level immunity above the 80% protective threshold against outbreaks. We evaluated the long-term persistence of neutralising antibodies to yellow fever virus following routine vaccination in infants aged around 9 months.

## Methods

### Study design and participants

We did a longitudinal cohort study. We studied two groups of healthy children: one from Ghana and one from Mali. Both groups are subsets of the study populations in trials of the meningococcal group A conjugate vaccine, MenAfriVac, administered to infants in association with other local Expanded Programme on Immunization vaccines, namely the PsATT-004 (phase 2, ISRCTN82484612) and Pers-004 (phase 4, ISRCTN10763234) studies in Ghana and the PsATT-007 (phase 3, PACTR201110000328305) and Pers-007 (phase 4, ISRCTN37623829) studies in Mali (appendix p 2).

The children were concomitantly vaccinated under the Expanded Programme on Immunization schedule against yellow fever and measles at 8–12 months of age. Ghanaian children received the YF-17DD yellow fever vaccine strain (Bio-Manguinhos-Fiocruz, Rio de Janeiro, Brazil), and Malian children were vaccinated with the YF-17D-213 strain (Chumakov Institute of Poliomyelitis and Viral Encephalitides of the Russian Academy of Sciences, Moscow, Russia). The children provided serum samples either around 2·3 and 6·0 years (PsATT-004 and Pers-004, Ghana) or 4·5 years (Pers-007, Mali) after vaccination (table 1). Only healthy children with available day-0 sera, a complete follow-up history, and no record of yellow fever revaccination were included; children who were seropositive for yellow fever at baseline were excluded.

**Table 1:** Schedule of sample collections after yellow fever vaccination

	**Time elapsed since vaccination**			**Age at sample collection**		
	Median, years	Range, days	Median (IQR), days	Range, months	Median (IQR), months	Mean (SD), years
Ghana, year 2·3	2·3	733–874	823 (815–830)	36–38	36 (36–36)	3·0 (0·01)
Ghana, year 6·0	6·0	2048–2331	2180 (2142–2219)	77–85	80 (79–82)	6·7 (0·1)
Mali, year 4·5	4·5	1444–1793	1626 (1514–1688)	56–68	62 (59–64)	5·2 (0·3)

The study protocol was approved by the ethics committees for studies involving human participants at the Navrongo Health Research Centre, Navrongo, Ghana, and the National Institute for Research in Public Health, Bamako, Mali. All procedures complied with the Declaration of Helsinki. Written informed consent was provided by the participants' parents or legal guardians in accordance with international ethical guidelines for epidemiological studies and with applicable local ethical guidance and requirements.

### Procedures

Blood samples were obtained by standard venipuncture. The measured outcomes were the serum concentration of neutralising antibodies to yellow fever virus 4·5 years after vaccination (Mali) or 2·3 and 6·0 years after vaccination (Ghana) and, in each cohort, the relative proportions of seropositive, borderline, and seronegative study participants at these timepoints. Titres of neutralising antibodies to yellow fever virus were determined by a microneutralisation assay. Briefly, 100 50% tissue culture infective doses of a yellow fever virus suspension (strain YF-17D-204, Stamaril, Sanofi Pasteur, Val de Reuil, France) were reacted with serial two-fold dilutions of sera (starting at 1:4) before inoculation into Vero cells cultured in 96-well plates, which were microscopically examined for cytopathic effect after 7 days. Baseline samples were tested in the same run as the matching post-vaccination sera. A neutralisation antibody titre of 1:10 or higher is considered a surrogate of protection.^8^, ^9^ However, to facilitate comparisons with antibody data collected by others using non-equivalent methods, we converted our titres to standardised concentrations in IU/mL by including in every assay two standard samples for yellow fever neutralising antibodies, which were themselves calibrated at 426·82 IU/mL and 106·70 IU/mL with reference to the First International Standard for yellow fever vaccine (WHO International Standard, NISBC 99/616) reconstituted at 143·00 IU/mL. On the basis of earlier studies,^10^, ^11^, ^12^ we applied a concentration threshold to discriminate seropositive (≥0·5 IU/mL) from borderline sera (measurable concentrations <0·5 IU/mL). Accordingly, we defined seroconversion and seroreversion as the crossing of this threshold over time. We classified samples as seronegative if they were ineffective at the initial dilution in the assay (1:4).

### Statistical analyses

By calculating the bounds of the 95% CI by the asymptotic method assuming 50% seropositivity, we determined that a sample of 386 vaccinees or more would constrain the 95% CI for the percentage of seropositive participants to a 5% interval around the empirical percentage.

We analysed the two vaccinee samples from Ghana and Mali separately. We used χ^2^ tests to compare the proportions of boys and girls between two immunogenicity strata, Boschloo's exact unconditional test^13^ for other 2 × 2 comparisons between independent groups, and McNemar's test on the proportions for dichotomous outcomes (eg, positives and negatives) in paired sera.

We characterised sets of vaccinees by the geometric mean, the median, and the IQR of antibody concentrations. We used a percentile bootstrap procedure with 10^6^ replications to compute the 95% CI for the geometric mean. We compared two sets of concentrations by either an independent-samples permutation test on the difference of geometric means or a permutation-based sign test for paired data, as applicable. The permutation tests involved 999 999 random replications, ensuring precision to three decimal digits for the p value near the significance threshold, as estimated by the 95% CI for the p value.^14^ Further details on the tests are provided in the appendix (p 3).

We did statistical analysis with R (version 3.4.3 and version 3.5.3) software. Hypothesis tests were two-sided, and the significance threshold was set at α=0·05.

### Role of the funding source

The funders of the study had no role in study design, data collection, data analysis, data interpretation, or writing of the report. CD and JEM had full access to all the data in the study. The corresponding author had final responsibility for the decision to submit for publication.

## Results

We included 436 children (226 [52%] boys, 210 [48%] girls) from rural communities in northern Ghana who received yellow fever vaccination as part of the Expanded Programme on Immunization between June 5, 2009, and Feb 25, 2010, and 587 children (297 [51%] boys, 290 [49%] girls) from urban communities in Mali who received vaccination between March 6, 2012, and Dec 26, 2012. The mean age at which children were vaccinated was 9·0 months (SD 0·3) in Ghana and 9·4 months (0·8) in Mali. The children provided blood samples 2·3 and 6·0 years after vaccination in Ghana and 4·5 years after vaccination in Mali (table 1).

**Figure 1:**
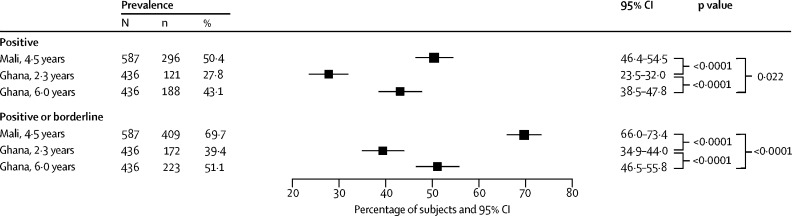
Forest plots of the prevalence of seropositive children Seropositive participants at the antibody concentration threshold of 0·5 IU/mL or more are shown at the top of the figure, and seropositive and borderline study participants are presented at the bottom as a merged category including all participants with a measurable titre. p values test the differences of proportions between groups.

In the Malian sample, 296 (50·4%; 95% CI 46·4–54·5) of 587 children were seropositive 4·5 years after vaccination (figure 1). Additionally, we classified 113 (19·3%, 95% CI 16·1–22·4) children as borderline (antibody concentration <0·5 IU/mL). When we merged positive and borderline strata into a broadly positive category, 409 (69·7%, 95% CI 66·0–73·4; table 2) were seropositive. This proportion presents a substantial drop from the overall number of children who seroconverted (290 [96·7%] of 300 children) 4 weeks after vaccination in data previously reported by others for different samples of the same vaccinee population.^15^

**Table 2:** Antibody concentration strata for participants with measurable neutralising antibodies

		**Participants**		**Neutralising antibodies (IU/mL)**	
		n (%, 95% CI)	Percentage of seropositive participants	Geometric mean (95% CI)	Median (IQR)
**Seropositive participants (antibody concentration ≥0·5 IU/ml)**					
Mali, year 4·5, n=587					
	Total	296 (50·4%, 46·4–54·5)	100·0%	1·120 (1·040–1·210)	0·915 (0·702–1·440)
	Low tier	242 (41·2%)	81·8%	0·863 (0·826–0·902)	0·882 (0·610–1·210)
	High tier	54 (9·2%)	18·2%	3·530 (3·100–4·070)	2·890 (2·420–4·640)
Ghana, year 2·3, N=436					
	Total	121 (27·8%, 23·5–32·0)	100·0%	1·380 (1·170–1·660)	1·020 (0·721–2·030)
	Low tier	90 (20·6%)	74·4%	0·854 (0·792–0·922)	0·765 (0·610–1·210)
	High tier	31 (7·1%)	25·6%	5·560 (4·110–7·790)	4·080 (2·890–8·160)
Ghana, year 6·0, n=436					
	Total	188 (43·1%, 38·5–47·8)	100·0%	1·790 (1·550–2·080)	1·440 (0·854–2·870)
	Low tier	110 (25·2%)	58·5%	0·915 (0·856–0·978)	0·911 (0·647–1·210)
	High tier	78 (17·9%)	41·5%	4·610 (3·810–5·670)	2·890 (2·350–7·800)
**Borderline participants (measurable antibody concentration <0·5 IU/ml)**					
Mali, year 4·5		113 (19·3%, 16·1–22·4)	NA	0·335 (0·318–0·353)	0·361 (0·271–0·453)
Ghana, year 2·3		51 (11·7%, 8·7–14·7)	NA	0·359 (0·337–0·382)	0·381 (0·312–0·430)
Ghana, year 6·0		35 (8·0%, 5·5–10·6)	NA	0·334 (0·309–0·361)	0·361 (0·302–0·383)
**Broadly seropositive participants (all participants with a measurable antibody concentration)**					
Mali, year 4·5		409 (69·7%, 66·0–73·4)	NA	0·801 (0·742–0·865)	0·721 (0·457–1·220)
Ghana, year 2·3		172 (39·4%, 34·9–44·0)	NA	0·926 (0·796–1·090)	0·721 (0·455–1·400)
Ghana, year 6·0		223 (51·1%, 46·5–55·8)	NA	1·380 (1·190–1·600)	1·170 (0·640–2·360)

The low and high tiers of seropositive participants are differentiated at the 1·8 IU/ml or more threshold. NA=not applicable.

121 (27·8%; 95% CI 23·5–32·0) of 436 Ghanaian children were seropositive to yellow fever virus 2·3 years after vaccination (figure 1). Among the large majority below the seropositivity threshold, we classified 51 (11·7%, 8·7–14·7) as borderline (table 2). Combination of the positive and borderline strata resulted in 172 children (39·4%, 34·9–44; figure 1). As in Mali, the proportion of children with broad-sense seropositivity was much smaller than the overall proportion observed 4 weeks after vaccination (611 [72·7%] of 841 children tested) in a previous study of the same Ghanaian population.^15^

In summary, despite differences between our Mali and Ghana groups, these data show a substantial long-term decline in immunity relative to the early outcome of vaccination.

**Figure 2:**
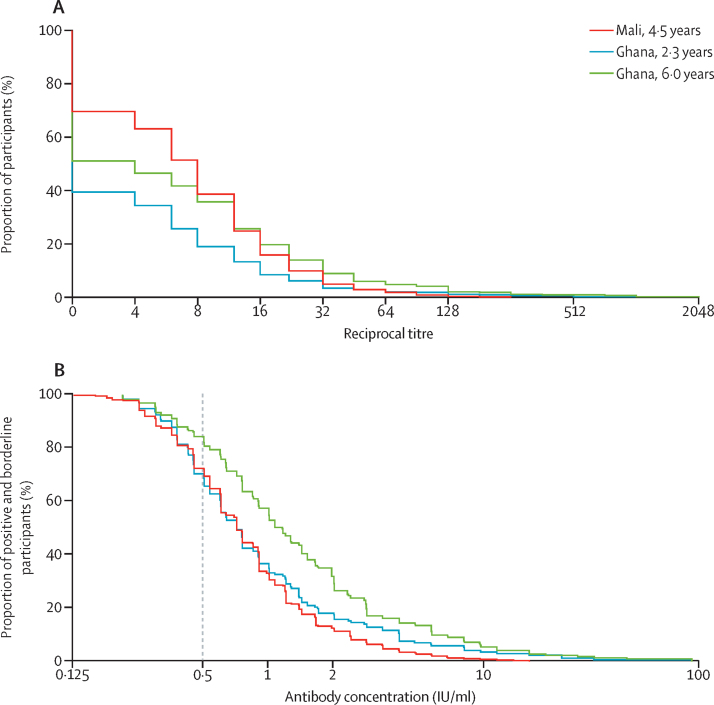
Reverse cumulative distribution plots of neutralising antibody values (A) Raw titres in the plaque reduction seroneutralisation assay for the full study groups. (B) Standardised antibody concentrations for broadly seropositive children (ie, seropositive and borderline study participants). The dotted line denotes the threshold for strict seropositivity at 0·5 IU/ml.

We found the prevalence of seropositivity to be greater among the Malian children despite the longer follow-up time (50·4% [95% CI 46·4–54·5] *vs* 27·8% [23·5–32·0]; Figure 1, Figure 2), but the prevalence after 4 weeks from an earlier study^15^ was also greater in the Malian than in the Ghanaian population samples. The distributions of measurable antibody concentrations (ie, those of broadly seropositive participants) in the Malian and the year-2·3 Ghanaian sera were similar, and we recorded the same median concentration (0·721 IU/mL), but we observed an excess of higher values in the Ghanaian relative to the Malian sera (figure 2B; appendix p 4). The geometric mean concentration for the seropositive stratum was accordingly higher in the Ghanaian group at 1·38 IU/mL (95% CI 1·17–1·66) versus 1·12 IU/mL (95% CI 1·04–1·21) in Mali (p=0·0087; table 2; appendix p 5).

The paired samples taken 2·3 and 6·0 years after vaccination showed the evolution of immunogenicity in the Ghanaian cohort. This group had a larger proportion of seropositive individuals 6·0 years after vaccination (43·1% [95% CI 38·5–47·8] *vs* 27·8% [23·5–32·0]; figure 1; table 2). Year-6·0 antibody concentrations were also higher overall than the Ghana year-2·3 and the Mali year-4·5 values (figure 2B; appendix p 4). 94 Ghanaian children were serostable over the intervening period, representing 77·7% of the year-2·3 positives and 21·6% of vaccinees (appendix pp 5–6).

**Figure 3:**
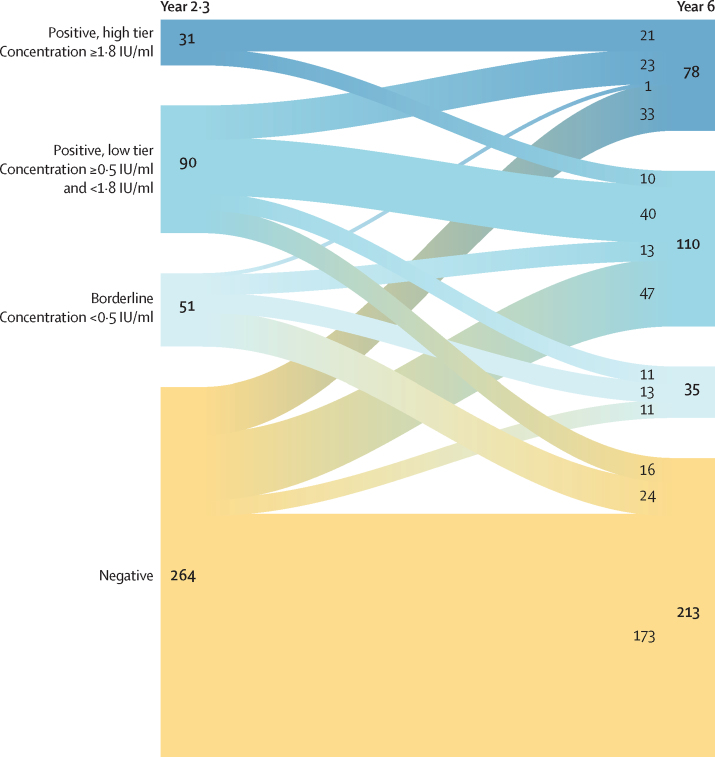
Evolution of the Ghanaian cohort Flow of study participants from a year-2·3 to a year-6·0 antibody concentration stratum. The thickness of the nodes (initial and final) and links between them are proportional to the number of participants as shown on the figure.

By contrast, 27 seropositive children (22·3% of seropositive children in year 2·3) seroreverted in the year-6·0 data—ie, deteriorated to negative or bordeline serostatus. These children had markedly lower year-2·3 antibody concentrations than the serostable children (geometric mean 0·74 IU/mL [95% CI 0·67–0·83] *vs* 1·65 IU/mL [1·35–2·05], p=0·0075; appendix pp 5, 7). We observed that lower year-2·3 concentrations were associated with higher year-6·0 seroreversion frequencies, and that study participants with values 1·8 IU/mL or more at year 2·3 were seroreversion-free (p<0·0001; appendix p 8). We consequently used 1·8 IU/mL as a tentative functional threshold to define a low and a high tier of seropositives. 27 (30%) of the 90 low-tier year-2·3 seropositives seroreverted at year 6·0 after vaccination, whereas an even larger proportion of year-2·3 borderline participants (24 [47%] of 51) deteriorated to negative serostatus (figure 3).

94 (29·8%) of the 315 year-2·3 seronegative or borderline children seroconverted between the two collection campaigns and accounted for 50% of the seropositive children in year 6·0 (figure 3; appendix p 7). Negative and borderline children seroconverted in the year-6·0 sample with similar frequencies (30·3% *vs* 27·0%, p=0·73; figure 3; appendix p 7). The proportions of low-tier and high-tier seropositive children in each group were, however, very different, with seroconversions of borderline participants markedly skewed toward the low tier (p=0·012; figure 3; appendix p 8).

Sex ratios in the immunity strata considered were unbiased relative to the source cohort, and we observed no differences by sex in antibody values (appendix p 9), except among serostable Ghanaian children, for whom the geometric mean concentration increased between the two follow-up points in boys, from 1·52 IU/mL (95% CI 1·15–2·05) to 2·18 IU/mL (95% CI 1·66–2·91; p=0·016), but not in girls (appendix pp 6, 10).

## Discussion

Since 2013, WHO has recommended a single dose of yellow fever vaccine for life-long immunity, and the amended International Health Regulations (2005) no longer require revaccination every 10 years.^5^, ^16^ Although it followed evidence of long-term immunogenicity,^17^, ^18^ this policy change has been controversial.^19^, ^20^, ^21^, ^22^ The one-dose recommendation bypasses the question of the duration of protective immunity elicited in infants, the target population of immunisation programmes in endemic countries.^6^ Relevant studies have focused on adults or older children, but the duration of immunity against yellow fever following infant vaccination has been studied only over short post-vaccination periods of 1–3 months.^23^ The scarcity of long-term studies on neutralising antibodies elicited in vaccinated infants has been identified as a knowledge gap in regard to the need for booster doses of the yellow fever vaccine.^23^

Studies have shown a progressive, long-term decrease in neutralisation titres in adults vaccinated against yellow fever (by 30%–40% within 5–10 years after vaccination),^6^ but also that children seroconvert at comparatively lower rates and develop weaker antibody titres.^17^ Waning seroprotection is a major determinant of the need for, and periodicity of, revaccination, and the population immunity achievable with a single dose of vaccine depends on the rate of vaccination failure and on the duration of conferred immunity. These parameters are important for immunisation programmes in endemic areas, where protective immunity covering about 80% of the population is necessary to prevent outbreaks.^1^ Forecasts of population coverage under the 2017–26 Eliminating Yellow Fever Epidemics strategy,^24^ which implements vaccination in infants as part of the Expanded Programme on Immunization in endemic countries, suggest that targets will not be met if conferred immunity is short-lived.

We have analysed the long-term persistence of neutralising antibodies against yellow fever virus among two population samples of African children vaccinated under the Expanded Programme on Immunization. The importance of humoral immunity in protecting against yellow fever is well established,^6^ as is the detection of neutralising antibodies (titre ≥1:10) as a surrogate for protective immunity. The role of T-cell-dependent immunity is uncertain; humoral immunity is known to be crucial, but neither the cellular component nor the presence of neutralising antibodies alone might be sufficient for protective immunity.^25^

We normalised assay results to a WHO international standard for yellow fever neutralising antibodies, which makes it possible to compare our data with those from other investigations using standardised antibody concentrations. Additionally, we conservatively introduced a seropositivity cutoff (≥0·5 IU/mL), because very low antibody concentrations might straddle the sensitivity threshold of the assay and were associated with inconsistent protection in previous studies.^26^, ^27^

Our findings point to a large decline in yellow fever immunity in children vaccinated as infants. The reduction in seropositivity, especially among the Ghanaian group after only 2·3 years, is noteworthy in comparison with previously reported seroconversion 28 days after vaccination.^15^ This suggests a rapid waning of immunity during the early years after infant vaccination, similar to the decline suggested to occur in adults during the first 5 years^6^, ^8^ but affecting a greater proportion of individuals or, conceivably, with faster kinetics. Additionally, half of the Ghanaian borderline participants became seronegative at 6·0 years, which suggests that Malian borderline participants (38·8% of borderline and seronegative participants) were likewise affected by waning immunity (eventual seroreversion could not be determined without a later follow-up sample).

Antigenic interference between the live-virus vaccines against measles and yellow fever co-administered under the Expanded Programme on Immunization should be considered as an explanation for the drop in immunity. In some studies, the immunogenicity of the yellow fever vaccine was decreased by co-administration with the measles vaccine, either alone^28^ or as part of the measles, mumps, rubella combination.^29^ In other studies,^30^, ^38^, ^39^ however, no interference could be shown. The sharp deterioration of immunity to yellow fever in our data shows that the effect of infant co-vaccination on the long-term evolution of immunogenicity needs to be addressed specifically.

The differences in seropositivity between the Ghanaian and Malian groups might owe less to the respective vaccine strains, which are genetically stable,^6^ than to factors such as vaccine dosage and handling,^31^ ethnic background,^32^ nutritional status, a seasonal effect on immunisation,^33^ exposure to other flaviviruses, or the immune microenvironment.^34^ A larger proportion of seroconverted individuals was determined for different samples from the same study populations (96·7% in Mali and 72·7% in Ghana) 28 days after vaccination in a previous study.^15^ Relative to the seropositivity in that study (at the ≥1:8 titre threshold), we noted an approximately two times long-term decrease in seropositivity for either population. Direct comparison, however, is hindered by differences in the population samples and study methods and by non-standardisation of antibody values in that earlier work.

The numerous late seroconversions in the Ghanaian year-6·0 data might have arisen from an external cause. The spread of a yellow fever outbreak in the Builsa, Kassena-Nankana West, and Kintampo South districts of Ghana in 2011 overlapped with the catchment area of this study and, chronologically, with the dates of the year-2·3 collection. Additionally, mass vaccination campaigns in the affected areas targeted 5·8 million people in early 2012.^35^ Vaccine immunity might have been boosted by natural yellow fever virus infection, unreported revaccination, or both. In this scenario, averted antibody decay in some children or new seroconversions in others could lead to an overestimation of the seropositivity resulting from the vaccine received at 9–12 months of age, a common issue in studies of vaccination in endemic areas.^6^ Unreported infection with wild or vaccinal virus might also have contributed to the enrichment in high antibody concentrations in the Ghanaian relative to the Malian seropositive children, and to the observed increase in year-6·0 antibody values among serostable boys. Although a higher antibody response to yellow fever vaccination has been noted previously in male individuals, the incidence of yellow fever is also greater in male than in female individuals, possibly determined by epidemiological and host-intrinsic factors.^36^ Additionally, the seroconversion of the majority of borderline participants to the low tier of seropositivity is in line with reports of a negative correlation between pre-existing antibodies against yellow fever and the magnitude of the B-cell response on subsequent exposure to the virus.^8^, ^34^, ^37^

The inability to control for environmental factors that might bias the measured response to yellow fever vaccination is a limitation of this study. We cannot exclude immune activation by unrelated infections or immunisations within the time frame of sample collection, or an antigenic challenge of memory immunity by other cross-reactive, circulating flaviviruses. Additionally, the presence of other vaccines, notably against measles, prevents the outcome of yellow fever vaccination from being assessed in isolation; however, it is unavoidable in a study in endemic countries with routine immunisation programmes, and the data are useful within this real-life context. The absence of two long-term follow-up points in Mali matching those of the Ghanaian cohort also limits comparisons between the two groups.

Our study shows that the long-term outcome of the vaccination of 9-month-old infants against yellow fever is unsatisfactory and argues for the one-dose-for-life recommendation to be reconsidered for this population in endemic countries. The beneficial effect of a booster dose has been established in individuals with low or negative neutralisation titres,^8^ but little evidence focuses on children. Further studies will be necessary to plan an ideal revaccination strategy against yellow fever within the wider schedule of childhood immunisations. A booster dose of vaccine would help to attain appropriate population immunity against yellow fever outbreaks.

## Data sharing

The anonymised data have been deposited in Zenodo and can be downloaded from the time of publication.
