# Supplementary material for: Long-term immunity against yellow fever in children vaccinated during infancy: a longitudinal cohort study
Source: Lancet Infect Dis. 2019 Dec;19(12):1363–70. doi: 10.1016/S1473-3099(19)30323-8 (PMC6892259; doi:10.1016/S1473-3099(19)30323-8)
Supplement: Supplementary appendix [file mmc1.pdf]

THE LANCET Infectious Diseases

Supplementary webappendix

This webappendix formed part of the original submission and has been peer reviewed. We post it as supplied by the authors.

Supplement to: Domingo C, Fraissinet J, Ansah PO, et al. Long-term immunity against yellow fever in children vaccinated during infancy: a longitudinal cohort study. *Lancet Infect Dis* 2019; published onlined Sept 19. https://doi.org/10.1016/S1473-3099(19)30323-8.

Appendix

Long-term immunity against yellow fever in children vaccinated during infancy: a longitudinal observational study

Contents

	Page
**Appendix note 1:** References for the MenAfriVac studies in international trial registries	2
**Appendix note 2:** Permutation tests	3
**Appendix figure 1:** Density plots of the distributions of antibody concentrations	4
**Appendix figure 2:** Box plots of antibody concentrations in categories of seropositive children	5
**Appendix table 1:** Statistics for serostable participants in the Ghana cohort	6
**Appendix table 2:** Statistics for seroreverters and late seroconverters in the Ghana cohort	7
**Appendix figure 3:** Seroreversion and late seroconversion in the Ghanaian group	8
**Appendix table 3:** Proportions of males and females in the study populations and serological strata	9
**Appendix table 4:** Mean antibody concentrations by sex and seropositivity tier	9
**Appendix table 5:** McNemar's tests on the paired year-2·3 and year-6 sera from the Ghana cohort	10
**Appendix references**	10

Appendix note 1: References for the MenAfriVac studies in international trial registries

1. PsATT-004, Ghana.
   ISRCTN registry number ISRCTN82484612.
   URL: https://doi.org/10.1186/ISRCTN82484612

A phase II, double-blind, randomised, controlled, dose ranging study to evaluate the safety, immunogenicity, dose response and schedule response of a meningococcal A conjugate vaccine administered concomitantly with local expanded program on immunisation (EPI) vaccines in healthy infants.

2. Pers-007 (phase IV), Ghana.
   ISRCTN registry number ISRCTN10763234.
   URL: https://doi.org/10.1186/ISRCTN10763234

Evaluation of antibody persistence in Ghanaian children more than five years after vaccination with MenAfriVac® widely used in Sub-Saharan Africa to prevent epidemic meningitis.

3. PsATT-007, Mali.
   Pan African Clinical Trials Registry number PACTR201110000328305
   URL: https://pactr.samrc.ac.za/TrialDisplay.aspx?TrialID=328

A Phase III, double-blind, randomized, controlled study to evaluate the immunogenicity and safety of different schedules and formulations of a meningococcal A conjugate vaccine administered concomitantly with local EPI vaccines in healthy infants and toddlers.

4. Pers-007 (phase IV), Mali.
   ISRCTN registry number ISRCTN37623829
   URL: https://doi.org/10.1186/ISRCTN37623829

Long-term follow-up of children who participated at 9-12 months of age in clinical trial PsA-TT-007 in Mali.

Appendix note 2: Permutation tests

The permutation tests were designed after Good.[1] The R scripts implementing these tests are available at the URL: https://doi.org/10.5281/zenodo.2684194

Independent-samples permutation test

We performed a permutation test on two independent antibody concentration sets x and y of size n and m to test the null hypothesis that the geometric mean concentrations (GMCs) are the same between the two groups of study participants. For this, we computed the difference of the respective GMCs, *ΔGMC*. Next, we compared this statistic against an empirical null distribution of the difference of GMCs that we generated by the Monte Carlo approach, as follows. We combined x and y into a single dataset (under the null hypothesis that the two sets of observations are identically distributed), drew a randomly-permuted dataset of size $N = n + m$ by sampling the combined dataset without replacement, and split this permuted dataset into two subsets x' and y' of size n and m. We computed and recorded the difference of GMCs between x' and y', *ΔGMC'*, and repeated the sampling and computation steps for B = 999999 total replications. Lastly, we computed a p-value by the formula:

$$p = (T + 1)/(B + 1) \tag{1}$$

where T denotes the number of cases in the permutation distribution where *ΔGMC'* ≥ *ΔGMC* in absolute value (two-tailed test).

Paired-samples permutation test

We performed a paired-samples permutation test to assess net changes in antibody concentrations between the first and second serum collections from the same study group of size n. Specifically, we tested the null hypothesis that the sum of differences between the paired concentrations equaled zero. We log-transformed the concentration data, and computed the sum of differences S across the n pairs of values. Next, we took the absolute value of each difference, gave it a plus or minus sign at random, and computed and recorded the sum of the n randomly-signed differences, S'. We repeated this step for B = 999999 total replications to build an empirical null distribution for the sum of differences. Lastly, we computed a p-value using equation (1) above with T as the number of cases in the permutation distribution where $S' \geq S$ in absolute value (two-tailed test).

Choice of the number of permutations

B = 999999 was selected as sufficient to provide precision to three decimal digits when $p = 0{\cdot}05$, i.e. such that the 95% confidence interval (CI) for the true p-value lie inside the interval [0·0495, 0·0505]. The bounds of the 95% CI for the true p-value were computed by the following formula after Ruxton and Neuhäuser,[2]

$$p \pm z_{(1-0\cdot5\alpha)}\sqrt{\frac{p(1-p)}{B+1}} \tag{2}$$

where $z_{(1-0\cdot5\alpha)}$ denotes the $(1 - 0{\cdot}5\alpha)$ quantile of the standard normal distribution (≈ 1·96 for α = 0·05), and p = 0·05.

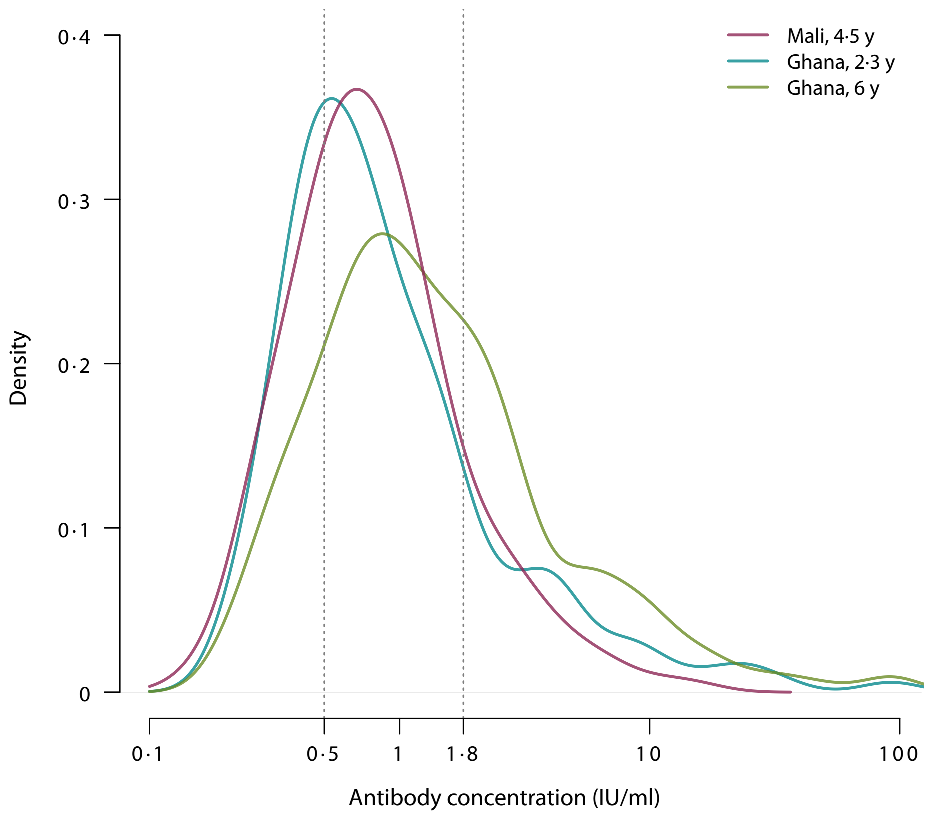

Appendix figure 1: Kernel density plot of the distributions of antibody concentrations. The figure compares the distributions of antibody concentrations between study groups and follow-up time points. The data correspond in each case to the combined seropositive and borderline strata. The dotted lines denote the seropositivity threshold (0·5 IU/ml), and the 1·8 IU/ml threshold between the low and high tiers of seropositives.

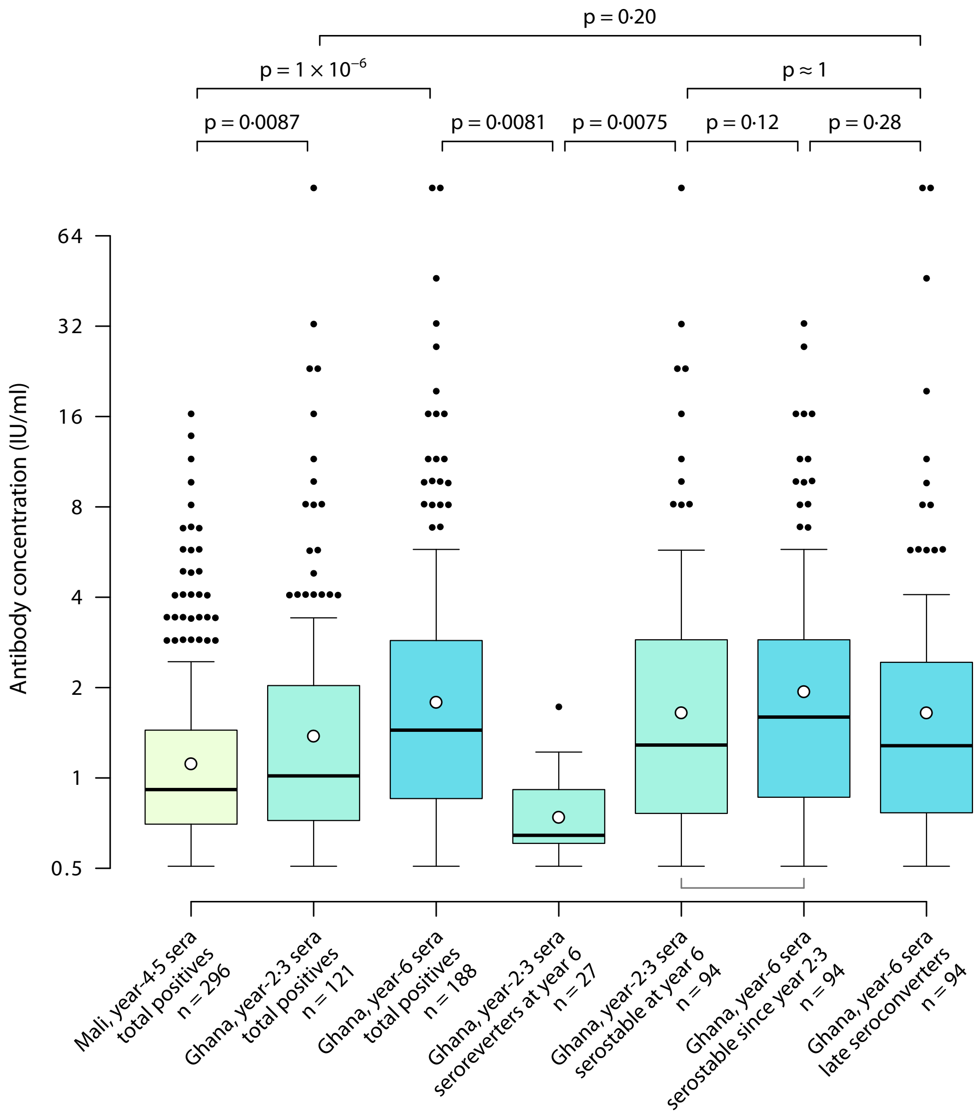

Appendix figure 2: Box plots of antibody concentrations in categories of seropositive children. White dots denote the respective geometric mean concentrations (GMCs); paired data sets are connected by an underside bracket. Two-tailed p-values test for equality of GMCs (independent-samples permutation test) or a net concentration change in the paired samples (permutation sign-test).

I. Serostable participants	N	Percentage of vaccinees (95% CI)	Percentage of year-2·3 seropositives	Percentage of year-6 seropositives	Males (%)	Females (%)
Total	94	21·6% (17·7–25·4)	77·7%	50·0%	49 (52%)	45 (48%)
Low tier, year 2·3	63	14·4	52·1%	n.a.	36 (57%)	27 (43%)
High tier, year 2·3	31	7·1	25·6%	n.a.	13 (42%)	18 (58%)
Low tier, year 6	50	11·5	n.a.	26·6%	22 (44%)	28 /56%)
High tier, year 6	44	10·1	n.a.	23·4%	27 (61%)	17 (39%)

II. Antibody concentrations (IU/ml)	GMC (95% CI)	Median concentration (IQR)	GMC, males (95% CI)	GMC, females (95% CI)
Year-2·3 sera				
Total	1·65 (1·35–2·05)	1·29 (0·762–2·87)	1·52 (1·15–2·05)	1·81 (1·35–2·50)
Low tier	0·908 (0·827–0·997)	0·858 (0·684–1·29)	0·906 (0·798–1·03)	0·911 (0·797–1·04)
High tier	5·56 (4·12–7·78)	4·08 (2·89–8·16)	6·33 (3·97–10·4)	5·07 (3·50–8·07)
Year-6 sera				
Total	1·94 (1·60–2·38)	1·60 (0·873–2·89)	2·18 (1·66–2·91)	1·71 (1·31–2·27)
Low tier	0·931 (0·844–1·03)	0·910 (0·721–1·26)	0·920 (0·800–1·06)	0·940 (0·819–1·08)
High tier	4·47 (3·57–5·68)	3·41 (2·04–8·14)	4·41 (3·28–6·05)	4·58 (3·27–6·63)

Appendix table 1: Statistics for serostable participants in the Ghana cohort. The data paired by dotted-line brackets show an increase in antibody concentrations in year 6 among males but not among females. The low and high tiers of seropositive participants are differentiated at the ≥1·8 IU/ml threshold. CI, confidence interval; GMC, geometric mean concentration; IQR, interquartile range; n.a., not applicable.

I. Seroreversion	N	Percentage of vaccinees (95% CI)	Percentage of year-2·3 seropositives	Percentage of year-6 total seronegatives	From the low tier of seropositives	From the high tier of seropositives	Males (%)	Females (%)
Total seroreverters	27	6·2%	22·3%	10·9%	27	0	17 (63%)	10 (37%)
To seronegative	16	3·7%	13·2%	6·5%	16	0	10 (62%)	6 (38%)
To borderline	11	2·5%	9·1%	4·4%	11	0	7 (64%)	4 (36%)

	Year-2·3 antibody concentrations (IU/ml)		
	GMC (95% CI)	Median	IQR
Total seroreverters	0·741 (0·665–0·832)	0·644	0·605–0·915
From the low tier	0·741 (0·665–0·832)	0·644	0·605–0·915
From the high tier	n.a.	n.a.	n.a.
To seronegative	0·689 (0·613–0·778)	0·610	0·591–0·907
To borderline	0·823 (0·686–1·01)	0·762	0·607–0·965

II. Late seroconversion	N	Percentage of vaccinees (95% CI)	Percentage of year-2·3 non-seropositives	Percentage of year-6 total seropositives	From seronegative (percentage of seronegatives)	From bordeline (percentage of borderline)	Males (%)	Females (%)
Total seroconverters	94	21·6%	29·8%	50·0%	80 (30·3%)	14 (27%)	50 (53%)	44 (47%)
To the low tier	60	13·8%	19·0%	31·9%	47 (17·8%)	13 (25%)	36 (60%)	24 (40%)
To the high tier	34	7·8%	10·8%	18·1%	33 (12·5%)	1 (2%)	14 (41%)	20 (59%)

	Year-6 antibody concentrations (IU/ml)		
	GMC (95% CI)	Median	IQR
Total seroconverters	1·65 (1·35–2·06)	1·28	0·765–2·43
From seronegative	1·75 (1·40–2·24)	1·44	0·832–2·86
From borderline	1·16 (0·838–1·90)	1·02	0·763–1·23
To the low tier	0·901 (0·824–0·986)	0·911	0·643–1·19
To the high tier	4·80 (3·49–6·92)	2·88	2·37–5·77

Appendix table 2: Statistics for seroreverters and late seroconverters in the Ghana cohort. Seronegative denotes no read-out at the lowest dilution in the assay. Borderline, measurable antibody concentration <0·5 IU/ml. Seropositive, antibody concentration ≥0·5 IU/ml. Seroreversion denotes deterioration to the borderline or negative stratum; late seroconversion denotes amelioration of negative or borderline participants to the seropositive stratum 6 years postvaccination. The low and high tiers of seropositive participants are differentiated at the ≥1·8 IU/ml threshold. CI, confidence interval; GMC, geometric mean concentration; IQR, interquartile range; n.a., not applicable.

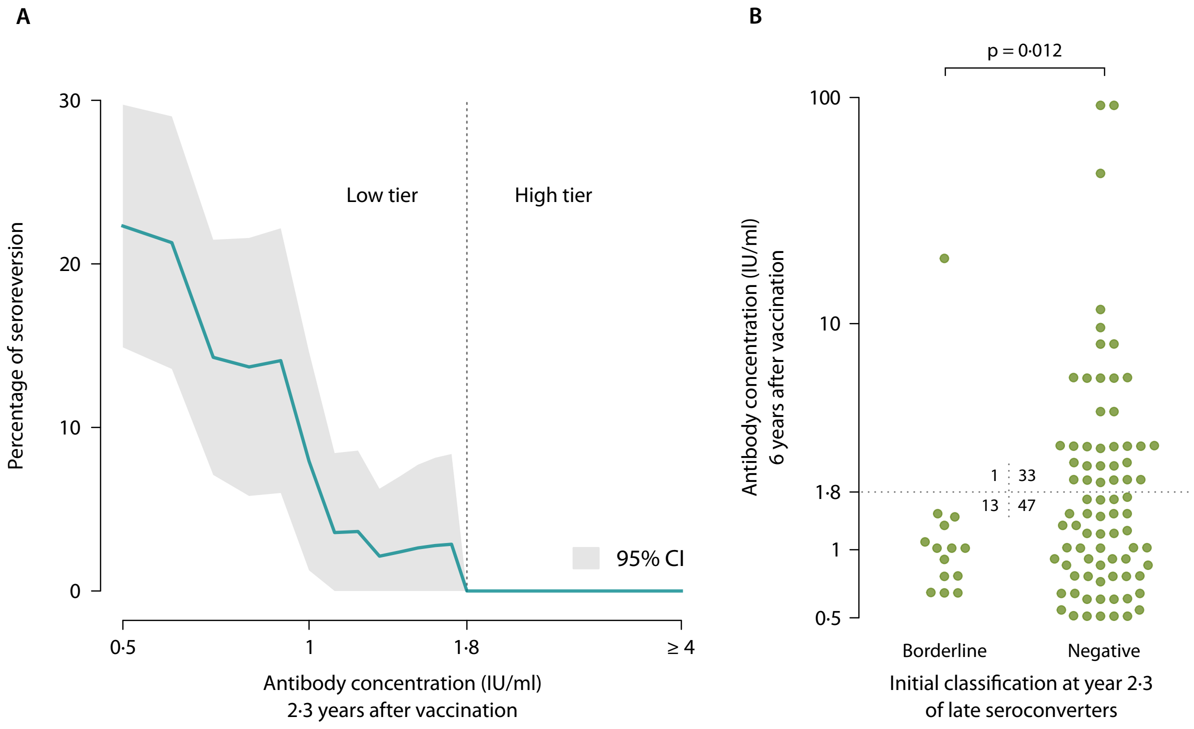

Appendix figure 3: Seroreversion and late seroconversion in the Ghanaian group. Seroreversion denotes deterioration to the borderline or negative stratum; late seroconversion denotes amelioration of negative or borderline participants to the seropositive stratum 6 years postvaccination. (**A**) Percentage of seroreverters as a function of a moving concentration threshold. The 1·8-IU/ml threshold (dotted line) defined a seroreversion-free high tier of seropositive participants. (**B**) Year-6 antibody concentrations for late seroconverters (n=94), split by year-2·3 source stratum; p-value from Boschloo's test of the proportions of low- and high-tier seropositives (inset).-

	Number of participants				
**Study population**	**Total**	**Males (%)**	**Females (%)**	**P-value**	**χ^2 test**
Mali, year 4·5	587	297 (50·6%)	290 (49·4%)	n.a.	n.a.
Ghana, year 2·3 and year 6	436	226 (51·8%)	210 (48·2%)	n.a.	n.a.
**A. Seropositive participants** (antibody concentration ≥0·5 IU/ml)					
Mali, year 4·5					
Total	296	151 (51%)	145 (49%)	0·91	1
Low tier	242	127 (52·5%)	115 (47·5%)	0·29	2
High tier	54	24 (44%)	30 (56%)		
Ghana, year 2·3					
Total	121	66 (54·5%)	55 (45·5%)	0·58	1
Low tier	90	53 (59%)	37 (41%)	0·14	2
High tier	31	13 (42%)	18 (58%)		
Ghana, year 6					
Total	188	99 (52·7%)	89 (47·3%)	0·83	1
Low tier	110	58 (52·7%)	52 (47·3%)	1	2
High tier	78	41 (53%)	37 (47%)		
**B. Borderline participants** (measurable antibody concentration <0·5 IU/ml)					
Mali, year 4·5	113	52 (46%)	61 (54%)	0·35	1
Ghana, year 2·3	51	27 (53%)	24 (47%)	0·89	1
Ghana 6 years	35	18 (51%)	17 (49%)	1	1
**C = A + B. Broadly seropositive participants** (all participants with a measurable antibody concentration)					
Mali, year 4·5	409	203 (49·6%)	206 (50·4%)	0·74	1
Ghana, year 2·3	172	93 (54·1%)	79 (45·9%)	0·59	1
Ghana, year 6	223	117 (52·5%)	106 (47·5%)	0·89	1

Appendix table 3: Proportions of males and females in the study populations and serological strata. χ^2 test 1: Tests the hypothesis that the proportions of males and females in a given serological stratum are the same as in the total study population. **χ^2 test 2:** Tests the hypothesis that the proportions of low- and high-tier seropositives are equal between males and females. The low and high tiers of seropositive participants are differentiated at the ≥1·8 IU/ml threshold. n.a., not applicable.

	Geometric mean concentration and 95% CI (IU/ml)		
**Study population**	**Males**	**Females**	**P-value**
**Seropositive participants** (antibody concentration ≥0·5 IU/ml)			
Mali, year 4·5	1·06 (0·964–1·17)	1·18 (1·05–1·33)	0·16
Low tier	0·858 (0·808–0·912)	0·869 (0·815–0·926)	0·79
High tier	3·20 (2·78–3·73)	3·82 (3·12–4·77)	0·23
Ghana, year 2·3	1·28 (1·03–1·62)	1·52 (1·17–2·02)	0·35
Low tier	0·863 (0·779–0·957)	0·842 (0·755–0·942)	0·77
High tier	6·33 (3·97–10·4)	5·07 (3·50–8·06)	0·51
Ghana, year 6	1·80 (1·46–2·24)	1·78 (1·47–2·18)	0·95
Low tier	0·876 (0·802–0·959)	0·960 (0·869–1·06)	0·19
High tier	4·97 (3·76–6·78)	4·25 (3·31–5·59)	0·45

Appendix table 4: Mean antibody concentrations by sex and seropositivity tier. The low and high tiers of seropositive participants are differentiated at the ≥1·8 IU/ml threshold. P-value from an independent-samples permutation test on the difference of geometric mean concentrations.

Comparison		Number of participants		Two-sided McNemar's test p-value	Odds ratio (95% CI)
		Year 6			
A	Year 2·3	Seronegative and borderline	Seropositive	7×10^{-10}	3·48 (2·25–5·56)
	Seronegative and borderline	221	94		
	Seropositive	27	94		
		Year 6			
B	Year 2·3	Seronegative	Seropositive and borderline	1×10^{-5}	2·28 (1·55–3·39)
	Seronegative	173	91		
	Seropositive and borderline	40	132		
	Females, year 2·3	Females, year 6			
C		Low tier	High tier	1	0·875 (0·27–2·76)
	Low tier	20	7		
	High tier	8	10		
		Males, year 6			
D	Males, year 2·3	Low tier	High tier	0·0013	8 (1·88–71·7)
	Low tier	20	16		
	High tier	2	11		

Appendix table 5: McNemar's tests on the paired year-2·3 and year-6 sera from the Ghana cohort. Seronegative denotes no read-out at the lowest dilution in the seroneutralization assay. Borderline, measurable antibody concentration <0·5 IU/ml. Seropositive, antibody concentration ≥0·5 IU/ml. The low and high tiers of seropositive participants are differentiated at the ≥1·8 IU/ml threshold. CI, confidence interval.

A. Cohort-wide seroconversion versus seroreversion events between years 2·3 and 6 post-vaccination. Seroconversion exceeds seroreversion.

B. Cohort-wide amelioration versus deterioration cases between years 2·3 and 6 post-vaccination. Similar to (**A**) above but merging together the seropositive and borderline strata. Amelioration exceeds deterioration.

C. Serostable females: upward transitions from the low to the high tier of seropositives, versus downward transitions from the high to the low tier, between years 2·3 and 6 post-vaccination. Upward and downward transitions are approximately balanced.

D. Serostable males: upward transitions from the low to the high tier of seropositives, versus downward transitions from the high to the low tier, between years 2·3 and 6 post-vaccination. Upward transitions exceed downward transitions.

Appendix references

1. Good P. Permutation tests: a practical guide to resampling methods for testing hypotheses. 2nd ed. New York: Springer-Verlag, 2000.
2. Ruxton GD, Neuhäuser M. Improving the reporting of P-values generated by randomization methods. *Methods Ecol Evol* 2013; **4:** 1033-6.
